# Autophagy‐Activated Self‐reporting Photosensitizer Promoting Cell Mortality in Cancer Starvation Therapy

**DOI:** 10.1002/advs.202301295

**Published:** 2023-04-21

**Authors:** Ruoyao Zhang, Chen Zhang, Chao Chen, Minggang Tian, Joe H. C. Chau, Zhao Li, Yuanzhan Yang, Xiaoqiong Li, Ben Zhong Tang

**Affiliations:** ^1^ School of Medical Technology Institute of Engineering Medicine School of Life Science Beijing Key Laboratory for Separation and Analysis in Biomedicine and Pharmaceuticals Beijing Institute of Technology Beijing 100081 P. R. China; ^2^ Department of Chemical and Biological Engineering and Department of Chemistry Hong Kong Branch of Chinese National Engineering Research Center for Tissue Restoration and Reconstruction Division of Life Science and State Key Laboratory of Molecular Neuroscience The Hong Kong University of Science and Technology Clear Water Bay, Kowloon Hong Kong P. R. China; ^3^ School of Chemistry and Chemical Engineering University of Jinan Jinan Shandong 250022 P. R. China; ^4^ School of Science and Engineering Shenzhen Institute of Aggregate Science and Technology The Chinese University of Hong Kong Shenzhen Guangdong 518172 P. R. China

**Keywords:** 3D tumor spheroid chip, autophagy‐activated photosensitizer, cancer starvation therapy, dual‐emissive self‐reporting AIEgen, photodynamic therapy

## Abstract

Cancer starvation therapy have received continuous attention as an efficient method to fight against wide‐spectrum cancer. However, during cancer starvation therapy, the protective autophagy promotes cancer cells survival, compromising the therapeutic effect. Herein, a novel strategy by combination of autophagy‐activated fluorescent photosensitizers (PSs) and cancer starvation therapy to realize the controllable and efficient ablation of tumor is conceived. Two dual‐emissive self‐reporting aggregation‐induced emission luminogens (AIEgens), TPAQ and TPAP, with autophagy‐activated reactive oxygen species (ROS) generation are prepared to fight against the protective autophagy in cancer starvation therapy. When protective autophagy occurs, a portion of TPAQ and TPAP will translocate from lipid droplets to acidic lysosomes with significant redshift in fluorescence emission and enhanced ROS generation ability. The accumulation of ROS induced by TPAQ‐H and TPAP‐H causes lysosomal membrane permeabilization (LMP), which further results in cell apoptosis and promotes cell death. In addition, TPAQ and TPAP can enable the real‐time self‐reporting to cell autophagy and cell death process by observing the change of red‐emissive fluorescence signals. Particularly, the efficient ablation of tumor via the combination of cancer starvation therapy and photodynamic therapy (PDT) induced by TPAQ has been successfully confirmed in 3D tumor spheroid chip, suggesting the validation of this strategy.

## Graphical abstract

Dual‐emissive self‐reporting AIEgens, TPAQ, and TPAP, have been constructed as autophagy‐activated photosensitizers to fight against the protective autophagy in cancer starvation therapy. When protective autophagy occurs, a portion of AIEgens translocate from lipid droplets to lysosomes with red‐shifted emission and enhanced ROS generation ability. The accumulation of ROS in lysosomes causes lysosomal membrane permeabilization, further promoting cancer cell mortality.

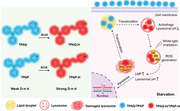

## 1 Introduction

Distinct from normal differentiated tissues, cancer cells must take up abundant glucose, amino acids, and lipids at an accelerated rate to support their non‐homeostatic proliferation. As such, blocking nutrient supply has been proposed as an effective and selective means to block cancer growth.^[^ ^1^, ^2^ ^]^ In recent years, cancer starvation therapy has attracted much attention for cancer treatment by cutting off the nutrient supply of tumor tissues to suppress their proliferation.^[^ ^3^, ^4^, ^5^ ^]^ However, under starvation, cancer cells would activate autophagy during which cancer cells can digest their own components to generate amino acids and free fatty acids for re‐establishing homeostasis. This protective autophagy sustains cancer cell growth, compromising the therapeutic effect of cancer starvation therapy.^[^ ^6^, ^7^, ^8^ ^]^ Therefore, it is an urgent need to inhibit the protective autophagy in cancer cells for cancer starvation therapy.

To avoid the protective autophagy in cancer starvation therapy, researchers mainly utilize autophagy inhibitors such as chloroquine (CQ) and hydroxychloroquine (HCQ) to regulate the autophagy process. For example, Li et al. have constructed CQ‐encapsulated biomimetic nanoparticles to achieve an enhanced anticancer effect.^[^ ^9^ ^]^ Qian et al. have developed iLipo‐H nanoparticles containing HCQ to inhibit autophagy by alkalizing lysosomes.^[^ ^10^ ^]^ However, the commonly used autophagy inhibitors like CQ and HCQ lack therapeutic selectivity. When CQ and HCQ diffuse from tumor site to normal tissues, they could also alkalize lysosomes, leading to physiological dysfunction of lysosomes in normal cells. Moreover, the detailed physiological and pathological information such as when the autophagy process occurs and when cell damage occurs is difficult to be monitored in real time using these autophagy inhibitors.

Photodynamic therapy (PDT) has emerged as the promising alternative approach for cancer treatment, since the PDT can achieve controllable ablation of tumor cells via the regulation of incident light.^[^ ^11^, ^12^, ^13^, ^14^, ^15^ ^]^ As the most important agentia in PDT, photosensitizers (PSs), especially the PSs based on organic fluorescent compounds, play vital roles in the efficacy of PDT, which could not only be activated under light irradiation to harvest toxic reactive oxygen species (ROS) to degrade tumors or cancerous masses, but their fluorescent signals can also be used for dynamically monitoring the tumor states.^[^ ^16^, ^17^ ^]^ Traditional organic fluorescent PSs, such as porphyrin derivatives, chlorins, and phthalocyanines often suffer from low tumor specificity, and the unspecific uptake in normal tissues brings in unnecessary side effects.^[^ ^18^, ^19^, ^20^ ^]^ Therefore, the activatable fluorescent PSs decorated with activable groups that could target the overexpressed proteins or other species in tumor‐specific microenvironment have been developed.^[^ ^21^, ^22^, ^23^, ^24^, ^25^ ^]^ However, the preparation of specific recognition elements against cancer cell receptors is complex and costly, and it is hard to achieve wide‐spectrum cancer treatment.

Herein we have conceived a novel strategy by the combination of autophagy‐activated PSs and wide‐spectrum cancer starvation therapy. The autophagy‐activated fluorescent PSs has been constructed, which would achieve the selective ablation of tumor cells owing to the high autophagy levels of cancer cells during the starvation therapy. When protective autophagy of cancer cells occurs, the fluorescent PSs could be switched on and generate ROS to fight against the protective autophagy, enhancing cancer starvation therapy. Meanwhile, the constructed fluorescent PSs stay inert in normal tissues. In detail, we have designed two dual‐emissive autophagy‐activated AIEgen‐based PSs, TPAQ and TPAP, to fight against the protective autophagy in cancer starvation therapy. Both TPAQ and TPAP could be responsive to acidic environment with the pKa values calculated as 3.51 and 4.01, respectively. In cancer cells under normal state, TPAQ and TPAP stain lipid droplets with blue emission and low ROS‐generated ability. While cancer cells undergo autophagy in starving state, a portion of TPAQ and TPAP molecules can migrate from lipid droplets to lysosomes with largely red‐shifted emission and simultaneously light‐driven ROS generation can also be activated. The excessive ROS induced by TPAQ‐H and TPAP‐H can lead to lysosomal membrane permeabilization (LMP), which blocked the protective autophagy and further caused cell apoptosis to promote cell death. After LMP occurred, TPAQ‐H and TPAP‐H can be leaked out resulting in attenuation of red emission in lysosomes. Moreover, the efficient ablation of tumor via the combination of cancer starvation therapy and PDT induced by TPAQ has been successfully confirmed in 3D tumor spheroid chip.

## 2 Results and Discussion

### 2.1 Design and Synthesis

**Scheme 1:**
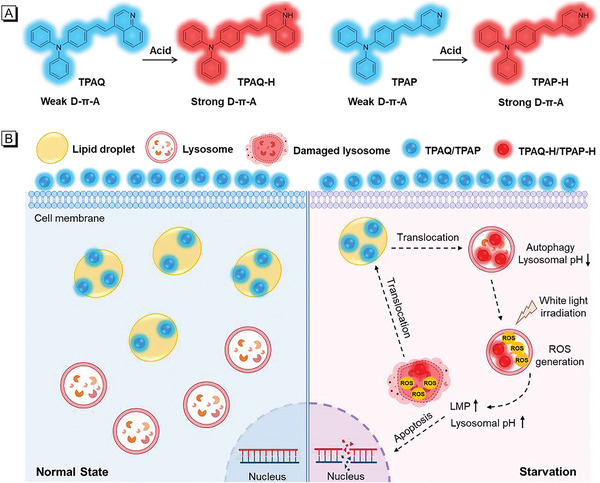
A) Chemical structures of TPAQ and TPAP in normal and acidic conditions. B) The schematic illustration of the effect mechanism of TPAQ and TPAP on A549 cells under normal and starving state.

In order to be activated by autophagy, the molecules to be designed should first respond to the acidic environment of lysosomes.^[^ ^26^ ^]^ In one of our previous works, we found an assistant molecule TPAQ that could stain lipid droplets with blue emission.^[^ ^27^ ^]^ From the chemical structure in **Scheme** **1**A, TPAQ is weakly basic because the nitrogen atom in quinoline group can be protonated in acid conditions. Similar to quinoline group, the nitrogen atom in the pyridine group should also be protonated in acid conditions, and thus another molecule TPAP is also be designed and synthesized in Scheme 1A. Since quinoline and pyridine groups are weak electron‐withdrawing group, both TPAQ and TPAP have weak donor‐*π*‐acceptor (D‐*π*‐A) structures. By measuring the *n*‐octanol/water partition coefficient (logP) shown in Figure S1 and Table S1 (Supporting Information), the logP value of TPAP was calculated as 1.79, indicating that TPAP was also lipophilic like TPAQ whose logP value was 3.71,^[^ ^27^ ^]^ and inclined to target lipid droplets. Due to the weak D‐*π*‐A structures of TPAQ and TPAP and low polarity of lipid droplets, we expected that TPAQ and TPAP first stain lipid droplets and emit blue fluorescence in living cells. When protective autophagy occurs in cancer starvation therapy, the pH value in lysosome decreases accompanied by the translocation of a portion of TPAQ and TPAP from lipid droplets to lysosomes. In lysosomal acidic environment, TPAQ and TPAP would be protonated to TPAQ‐H and TPAP‐H, respectively, with longer red emission and stronger ROS generation ability. Then under white light, abundant ROS accumulated into lysosomes disrupting lysosomal function to inhibit the protective autophagy. At the same time, the excessive ROS could also cause LMP, resulting in cell apoptosis and the translocation of TPAQ and TPAP from lysosomes to lipid droplets with decreased red emission. Therefore, TPAQ and TPAP could promote cell mortality in cancer starvation, and simultaneously, the staining position and fluorescence color changes of TPAQ and TPAP can reflect the different physiological state (Scheme 1B). The chemical structure of TPAQ has been characterized in our previous work,^[^ ^27^ ^]^ and the chemical structure of TPAP was fully characterized by ^1^H NMR, ^13^C NMR, and HRMS (Figure S9–S11, Supporting Information) in the ESI†.

### 2.2 Photophysical Properties

**Figure 1:**
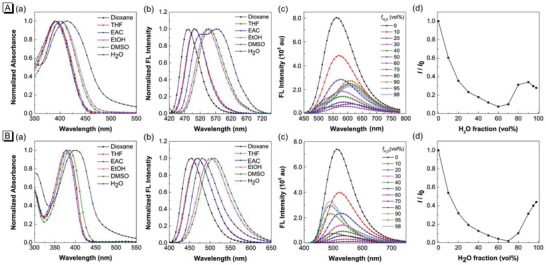
Normalized absorption a) and FL b) spectra of TPAQ (A) and TPAP (B) in different solvents; FL spectra c) of TPAQ (A) and TPAP (B) in EtOH and EtOH/H_2_O mixtures with different H_2_O fractions (*f*_H2O_); d) Changes in the FL peak intensities (*I*) of the solutions of TPAQ (A) and TPAP (B) with the H_2_O contents in the EtOH/H_2_O mixtures. *I*_0_ is the intensity in pure EtOH. Concentration: 10 µM.

The normalized absorption and fluorescence (FL) spectra of TPAQ and TPAP in different solvents are shown in **Figure** **1**Aa,b,Ba,b. TPAQ and TPAP displayed strong absorbance ≈400 and 380 nm, respectively. With the increase of solvent polarity, they showed bathochromic shift in fluorescence spectra due to twist intramolecular charge transfer (TICT) effect. The FL spectra of TPAQ and TPAP in EtOH and EtOH/H_2_O mixture with different H_2_O fraction are shown in Figure 1Ac,d,Bc,d. For TPAQ, with an increase in the H_2_O fraction from 0 to 60%, the emission decreased with a red shift in FL spectra, due to the enhancement of TICT effect. Further increasing the H_2_O fraction from 70% to 90%, the emission evidently increased with a red shift in FL spectra, showing AIE characteristics. When the H_2_O fraction was more than 90%, the FL intensity decreased a little bit, probably due to the change of the morphology and size of the aggregates. For TPAP, with the increase of H_2_O fraction, the emission intensity first decreased with red‐shift in FL spectra due to TICT effect. With the water content increasing from 70% to 98%, the emission of TPAP dramatically enhanced, indicating its AIE characteristics. Different from TPAQ, the FL spectra of TPAP blue‐shift when the H_2_O fraction was more than 70%, probably due to the different molecular packing mode in aggregate state.

**Figure 2:**
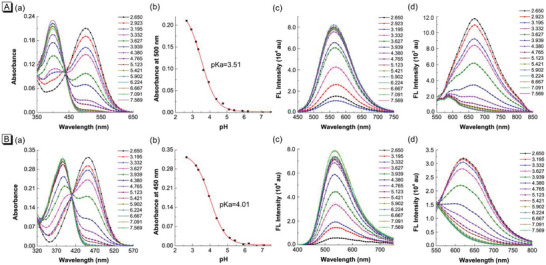
a) Absorption spectra of TPAQ (A) and TPAP (B) in EtOH/PBS (v:v = 1:1) solvents at different pH values; b) Plots of the absorbance of TPAQ (A) and TPAP (B) at different pH values and the fitted curve; c) FL spectra of TPAQ (A) and TPAP (B) excited at 400 and 380 nm, respectively; d) FL spectra of TPAQ (A) and TPAP (B) excited at 500 and 450 nm, respectively. Concentration: 10 µM.

To investigate whether TPAQ and TPAP could be responsive to acidic environment, the pH titration experiments with pH value range from 2.650 to 7.569 were carried out. Under the physiological state of normal pH (≈7.4), the main peaks in absorption spectra of TPAQ and TPAP were ≈400 and 380 nm, respectively, shown in **Figure** **2**a. When excited with 400 nm and 380 nm, respectively, the emission peaks of TPAQ and TPAP were ≈560 nm and 535 nm, showing green emission in Figure 2c. While in acidic conditions, the main peaks in absorption spectra of TPAQ and TPAP were red‐shifted to 500 and 450 nm, respectively, shown in Figure 2a. Under excitation at 500 nm and 450 nm, the emission peaks of TPAQ and TPAP were ≈670 nm and 620 nm, respectively, showing red emission in Figure 2d. For TPAQ, with the decrease of pH value, the absorption at 500 nm increased while the absorption at 400 nm decreased. The emission intensity ≈560 nm decreased while that at 670 nm increased. TPAP showed similar property with that of TPAQ. Based on the absorbance at 500 nm of TPAQ and the absorbance at 450 nm of TPAP, the pKa values were calculated as 3.51 and 4.01, respectively, shown in Figure 2b. Both the absorption and FL spectra of TPAQ and TPAP at different pH values indicated that the two molecules could be responsive to acidic environment. Particularly, they could be protonated in acidic environment and change the chemical structures form weak D‐*π*‐A to strong D‐*π*‐A (Scheme 1A), accompanied by obvious 110 and 85 nm redshifts in FL spectra of TPAQ and TPAP, respectively. Therefore, they were promising to serve as autophagy‐activated PSs that could be used in cancer starvation therapy.

### 2.3 In Vitro Cell Imaging

**Figure 3:**
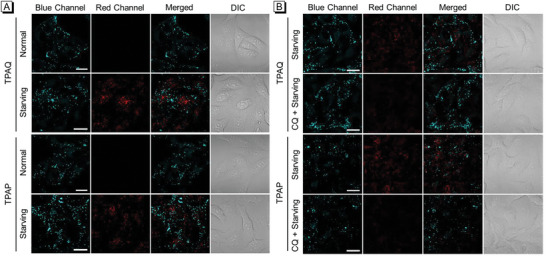
Confocal laser scanning microscopy (CLSM) images of live A549 cells stained with 2 µM TPAQ and TPAP under normal and starving conditions, respectively A), and under starving and CQ‐treated starving conditions, respectively B). Blue channel: *λ*_ex_ = 405 nm, *λ*_em_ = 410–480 nm; Red channel: *λ*_ex_ = 488 nm, *λ*_em_ = 600–700 nm. Scale bar = 20 µm.

Furthermore, the bioimaging experiments of TPAQ and TPAP were first performed in human lung cancer cells (A549). In **Figure** **3**A, it could be seen that normal A549 cells incubated with TPAQ or TPAP displayed intense blue emission and very weak red fluorescence. The dot‐like structures in cytoplasm in blue channel were observed clearly, which is typical structures of lipid droplets.^[^ ^28^ ^]^ Subsequently, co‐staining experiments with commercial lipid droplets’ probe Nile Red were carried out in Figure S2A (Supporting Information). The co‐localization coefficients of TPAQ and TPAP and Nile Red were both over 0.85, demonstrating the localization of TPAQ and TPAP in lipid droplets in normal A549 cells. Further to check whether TPAQ and TPAP could respond to cell's autophagy process, starvation‐induced autophagy experiments were carefully carried out. A549 cells were first stained with TPAQ and TPAP in complete culture medium, and then the culture medium was replaced by PBS buffer solution to induce autophagy.^[^ ^29^, ^30^ ^]^ In the starving groups of Figure 3A, in addition to the fluorescence signals in blue channel, strong fluorescence in cytoplasm in red channel were also detected. In the merged images, the blue emission showed little overlap with the red signal, which indicated that the staining position of partial TPAQ and TPAP changed after autophagy occurred. From the photophysical property measurement, it could be seen that the fluorescence of TPAQ and TPAP would undergo a significant red shift in an acidic environment. Considering that lysosomes are typical acidic organelles in cells, we speculated that a portion of TPAQ or TPAP migrated from lipid droplets to lysosomes during autophagy. To verify the speculation, co‐stain experiments were carried out to check the location of TPAQ and TPAP in the red channel. As shown in Figure S2B (Supporting Information), the staining pattern of TPAQ and TPAP were well merged with that of commercial lysosomal probe LysoTracker Deep Red (LTDR) under starving condition and the co‐localization coefficients were both over 0.70, indicating that TPAQ and TPAP located in lysosomes to give red fluorescence.

Afterward, we chose chloroquine (CQ), an inhibitor of autophagy that can alter the acidic environment of lysosomes, to further check whether TPAQ and TPAP could respond to cell autophagy. In Figure 3B, in the starving groups without CQ treatment, obvious fluorescence signals in red channel were observed. In contrast, in the cells pre‐treated with CQ then under starvation, very tiny fluorescence in red channel was detected, showing that TPAQ and TPAP would not translocate to lysosomes when autophagy was inhibited. Therefore, these results demonstrated that TPAQ and TPAP could well respond to cell autophagy and they would translocate from lipid droplets to lysosomes accompanied by a marked red shift in fluorescence.

### 2.4 ROS Generation Ability

**Figure 4:**
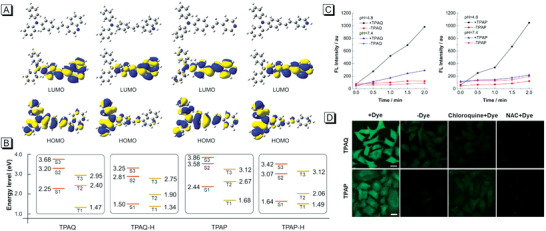
A) Optimized geometries and the frontier orbitals of TPAQ, TPAQ‐H, TPAP, and TPAP‐H; B) Diagram of energy level of singlet and triplet states of TPAQ, TPAQ‐H, TPAP, and TPAP‐H based on optimized singlet geometries; C) Changes of FL intensity at 535 nm of DCF‐DA in the presence or absence of TPAQ/TPAP in PBS with pH = 4.8 or pH = 7.4 under light irradiation for different time points; D) CLSM images of live A549 cells under starvation treated with “DCF‐DA + TPAQ/TPAP”, “DCF‐DA”, “DCF‐DA + chloroquine + TPAQ/TPAP”, “DCF‐DA + NAC + TPAQ/TPAP” with light irradiation for 5 min. Scale bar = 20 µm.

TPAQ and TPAP would be protonated as TPAQ‐H and TPAP‐H in lysosomes with acidic condition, which can change the electronic structure from weak D‐*π*‐A to strong D‐*π*‐A. To investigate the differences in ROS generation ability of these molecules theoretically, the density functional theory (DFT) calculations were first conducted using Gaussian 16 software package. As displayed in **Figure** **4**A, all the four molecules (TPAQ, TPAQ‐H, TPAP, and TPAP‐H) showed evidently different electron density distribution in HOMO and LUMO, indicating the intramolecular charge transfer upon excitation. The HOMO of the four molecules mainly distributed at the triphenylamine moiety, which was significantly transferred to the pyridine or quinoline parts in LUMO. Obviously, TPAQ‐H and TPAP‐H showed far larger changes than TPAQ and TPAP, which should be attributed to the increase of electron‐withdrawing property of the quinoline and pyridine after protonation. Furthermore, according to the previous reports, the energy gap (Δ*E*_st_) between S1 and T1 state is a key parameter for the intersystem crossing efficiency.^[^ ^31^, ^32^ ^]^ Small Δ*E*_st_ may result in high efficiency of intersystem crossing and facilitate the formation of T1 state to generate ROS. Therefore, the Δ*E*_st_ of the four molecules were calculated as illustrated in Figure 4B. The Δ*E*_st_ of TPAQ‐H and TPAP‐H was much smaller than that of TPAQ and TPAP, which demonstrated that the ROS generation of TPAQ‐H and TPAP‐H would be significantly higher than that of TPAQ and TPAP.

In addition to calculated results, the performance of TPAQ and TPAP to produce ROS in neutral and acid environments was also carefully measured. “2',7'‐dichlorodihydrofluorescein diacetate” (DCF‐DA) is a commercial indicator that is commonly used in accessing ROS activity.^[^ ^33^, ^34^ ^]^ For in vitro test, DCF‐DA was first hydrolyzed to a non‐fluorescent compound in alkaline buffer solution, which would be oxidized by ROS into DCF with strong emission ≈535 nm. In Figure 4C, with the extension of the irradiation time, the fluorescence intensity of DCF in acidic buffer solution increased more sharply than that in neutral buffer solution containing TPAQ or TPAP, indicating that ROS generation ability of TPAQ and TPAP in acid environment (i.e., TPAQ‐H and TPAP‐H) is much higher than that in neutral environment.

Subsequently, ROS generation efficiency of TPAQ and TPAP in A549 cells undergoing autophagy were investigated. We still utilized the method of replacing the complete medium with PBS buffer solution to starve the cells and induce autophagy. In Figure 4D; Figure S3 and S4 (Supporting Information), starving A549 cells cannot oxidize the DCF‐DA to highly green‐emissive DCF under light irradiation without TPAQ or TPAP. In contrast, after the addition of TPAQ or TPAP and light irradiation, strong fluorescence signals from DCF were observed and the signals enhanced with the extension of light irradiation, indicating that efficient ROS was generated in the starving A549 cells. When the cells were pretreated with CQ or N‐Acetyl‐L‐cysteine (NAC, a ROS scavenger),^[^ ^35^ ^]^ the fluorescence signals from DCF reduced apparently. These results demonstrated that when cancer cells underwent autophagy, the TPAQ‐H or TPAP‐H that translocated in lysosomes did generate efficient ROS under light irradiation.

### 2.5 Inducing Lysosomal Membrane Permeabilization

**Figure 5:**
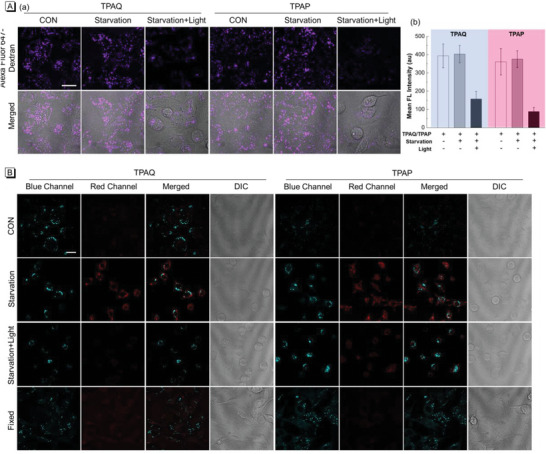
A) CLSM images of live A549 cells pre‐stained with 20 µM TPAQ and TPAP, respectively, and then treated with Alexa Fluor 647‐Dextran in “normal”, “starvation”, “starvation + light” conditions (a) and the relevant mean FL intensity of Alexa Fluor 647‐Dextran in a (b); for Alexa Fluor 647‐Dextran, *λ*_ex_ = 640 nm, *λ*_em_ = 650–720 nm; B) CLSM images of live A549 cells stained with TPAQ and TPAP, respectively, in “normal”, “starvation”, “starvation + light”, and “fixed” conditions; Blue channel: *λ*_ex_ = 405 nm, *λ*_em_ = 410–480 nm; Red channel: *λ*_ex_ = 488 nm, *λ*_em_ = 600–700 nm. Scale bar = 20 µm.

Efficient ROS in lysosomes could trigger LMP.^[^ ^36^, ^37^ ^]^ In order to testify whether the ROS induced by TPAQ‐H or TPAP‐H in lysosome could trigger LMP, we then performed the experiments to observe fluorescent dextran location. Generally, fluorescent dextran appears in punctate structures representing intact lysosomes in healthy cells, while after LMP fluorescent dextran would be released to cytosol and disappear from lysosomes.^[^ ^38^ ^]^ In **Figure** **5**Aa,b, in normal and starved A549 cells stained with TPAQ or TPAP, strong fluorescence signals and punctate pattern from Alexa Fluor 647‐Dextran were detected, indicating that lysosomal membrane was intact. While under light irradiation, the fluorescence signals dropped sharply, demonstrating that LMP occurred accompanied by Alexa Fluor 647‐Dextran release to cytosol. In order to further confirm that the decrease in fluorescence signals was caused by LMP rather than photobleaching effect, we tested the photostability of Alexa Fluor 647‐Dextran in cells. As shown in Figure S5 (Supporting Information), the mean fluorescence intensity between the experimental group and the control group displayed no obvious difference, indicating that the decrease in fluorescence signal was only caused by LMP. Therefore, these results showed that the rich ROS that was induced by TPAQ‐H or TPAP‐H in lysosomes could trigger LMP.

### 2.6 Self‐Reporting Autophagy and PDT Process

It should be noteworthy that when LMP occurred, TPAQ‐H and TPAP‐H could also leak out of lysosomes in addition to Alexa Fluor 647‐Dextran, resulting in a decrease in fluorescence signal of TPAQ‐H and TPAP‐H in lysosomes. Therefore, the fluorescence changes in red channel of TPAQ and TPAP could report the physiological state of cancer cells. Then we tested the fluorescence changes of the cells in different physiological states. As shown in Figure 5B, TPAQ could only stain lipid droplets in normal A549 cells, and thus only the fluorescence signals in blue channel were observed. When starvation triggered cell autophagy, a part of TPAQ translocated from lipid droplets to lysosomes with chemical structure changed to TPAQ‐H. The strong fluorescence from TPAQ‐H in red channel appeared. Subsequently under light irradiation, large amount of ROS induced by TPAQ‐H accumulated in lysosomes in the starved cells undergoing autophagy, which could trigger LMP. Then TPAQ‐H was leaked out from lysosomes, causing the evident decrease of fluorescence signals in red channel. In fixed cells (dead cells), the lysosomal membrane was also permeabilized, so that there was little fluorescence in red channel. Similar results were also obtained with TPAP. In order to further confirm that the fluorescence decrease in red channel was caused by LMP rather than photobleaching effect, the photostability of TPAQ and TPAP in acid conditions was tested under white light irradiation for 30 min. As shown in Figure S6 (Supporting Information), the fluorescence intensity of both TPAQ and TPAP in acid conditions displayed no obvious changes, indicating that fluorescence decrease in red channel was only caused by LMP. These results illustrated that TPAQ and TPAP could report the physiological state of cancer cells in autophagy and PDT process.

### 2.7 Countering Protective Autophagy and Inducing Cell Death

**Figure 6:**
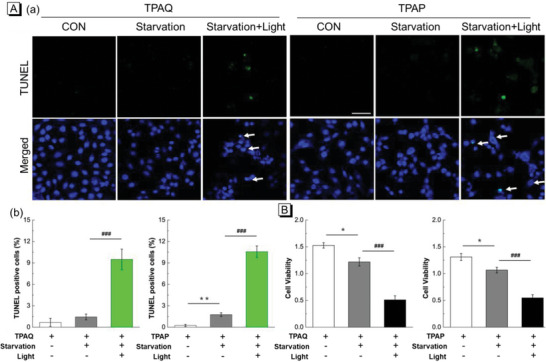
A) TUNEL staining of fixed A549 cells pre‐stained with 20 µM TPAQ and TPAP, respectively, in “normal”, “starvation”, and “starvation + light” conditions (a) and statistics of TUNEL positive cells (b) in relevant conditions in a; TUNEL: *λ*_ex_ = 488 nm, *λ*_em_ = 500–600 nm, DAPI: *λ*_ex_ = 405 nm, *λ*_em_ = 420–480 nm, Scale bar = 50 µm; B) Cell viability of A549 cells stained with 20 µM TPAQ and TPAP in “normal”, “starvation”, and “starvation + light” conditions. Power density: 50 mW cm^−2^. Bar graphs are presented as mean ± S.E. ^*^P<0.05, ^**^P<0.01 versus control group, ^###^P<0.001 versus no‐light group in unpaired two‐tailed t test.

Enhanced ROS cause LMP, resulting in the inhibition of protective autophagy and inducing cell apoptosis.^[^ ^37^ ^]^ Next TdT‐mediated dUTP nick end labeling (TUNEL) assay was carried out to investigate cell apoptosis caused by ROS produced by TPAQ‐H or TPAP‐H. As shown in **Figure** **6**Aa,b, in the case of only starvation without light‐irradiation‐generated ROS, cancer cells hardly underwent apoptosis. While under light irradiation with LMP occurring, the percentage of TUNEL positive cells increase significantly. These results indicated that ROS produced by TPAQ‐H or TPAP‐H in lysosomes indeed increased the apoptosis rate of cancer cells.

Subsequently, MTT (3‐(4,5‐dimethylthiazol‐2‐yl)‐2,5 diphenyl tetrazolium bromide) assay was performed to evaluate whether TPAQ and TPAP could further promote cell mortality in cancer starvation therapy. First, the effect of TPAQ and TPAP on cell viability of A549 cells under dark and “starvation + light” conditions were explored. As shown in Figure S7A,B (Supporting Information), TPAQ and TPAP at low doses (<20 µM) exhibited almost no cytotoxicity in cancer cells under normal state in dark conditions. While the cells were under “starvation + light” conditions, low doses of TPAQ and TPAP (< 20 µM) could cause significant reduction of cell viability in Figure S7C,D (Supporting Information). Then the cell viability of A549 cells stained with TPAQ and TPAP in “normal”, “starvation”, and “starvation + light” conditions was further compared. As shown in Figure 6B, compared with the control group, the cell viability in “starvation” group had a certain decrease, but in the group of “starvation + light”, the cell viability decreased significantly. In addition, the effect of TPAQ and TPAP on normal human fetal lung fibroblast‐1 (HFL‐1) cells, a cell line derived from normal tissues, was also evaluated. As shown in Figure S8 (Supporting Information), it could be seen that TPAQ and TPAP at a concentration of 20 µM barely damage normal cells under normal state, but TPAQ and TPAP at 20 µM would effectively ablate cancer cells at high autophagy levels. Therefore, these results demonstrated that TPAQ and TPAP could promote the cell mortality when cancer cells were in protective autophagy, but stay inert in normal cells.

### 2.8 Evaluation of PDT Effect in 3D Tumor Spheroid Chip

**Figure 7:**
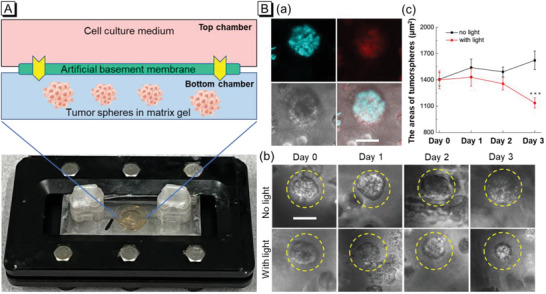
A) The physical object and schematic diagram of the tumor spheroid chip; B) CLSM images of A549 tumor spheres stained with 20 µM TPAQ in starving condition (a); bright field pictures of tumor spheres pre‐stained with 20 µM TPAQ in chip without or with white light irradiation in starving condition (b) and the statistics of the size of tumor spheres in b (c). Scale bar = 50 µm. Bar graphs are presented as mean ± S.E. ^***^P< 0.001 versus no‐light group in unpaired two‐tailed t test.

Compared with traditional bidimensional (2D) cell models, tridimensional (3D) cell models, such as spheroids, can simulate the features of solid tumors more accurately including spatial architecture and physiological responses.^[^ ^39^ ^]^ Particularly, tumor spheres can show more realistic response to antitumor drugs than that of 2D tumor cells. To further evaluate the PDT effect, by using microfluidic chip technology, we constructed a tumor spheroid chip as a drug screening platform to observe the response of tumor spheres to PSs. Since TPAQ has a redder fluorescence emission than TPAP, which is suitable for tissue imaging, we chose TPAQ to evaluate the PDT effect in the tumor spheroid chip. The physical object and schematic diagram of the tumor spheroid chip were shown in **Figure** **7**A. The cell culture medium filled the top chamber and A549 cells grew into tumor spheres within a few days in the matrix gel in the bottom chamber. The cell culture medium can enter the bottom chamber through the artificial basement membrane in the middle to provide energy for the cells. After stained with TPAQ, the culture medium was replaced with PBS buffer solution to induced autophagy. In Figure 7Ba, obvious fluorescence signals in blue and red channel were detected in tumor spheres undergoing autophagy. Then one tumor spheroid chip was irradiated with white light and the other one was kept in dark condition. We recorded the size of the tumor spheres in the two chips for three days. As shown in Figure 7Bb,c, in the chip of non‐irradiated group, the size of the tumor spheres increased to a certain extent, while in the other group, white light treatment exhibited obvious inhibition effect to tumor growth. These results indicated the PDT efficacy of TPAQ could efficaciously inhibit the growth of tumor spheres in autophagy, which was promising in cancer starvation therapy.

## 3 Conclusion

To summarize, we have successfully designed and synthesized two dual‐emissive self‐reporting AIEgens, TPAQ, and TPAP, as PDT sensitizers to fight against the protective autophagy in cancer starvation therapy. Both TPAQ and TPAP could be protonated in acidic environment with enhanced ROS generation ability, and thus the PDT effect of the two probes were activable after the lysosomal acidization upon autophagy. In living cells, TPAQ and TPAP could first target lipid droplets due to their lipophilic property to give blue emission. When cells underwent autophagy in starved state, a portion of TPAQ and TPAP would translocate to acidic lysosomes with largely red‐shifted emission, and simultaneously they could be protonated as TPAQ‐H and TPAP‐H with stronger ROS generation ability. The fluorescent dextran release experiments confirmed that the excessive ROS induced by TPAQ‐H and TPAP‐H caused LMP process. TUNEL assays certified that LMP further increased cell apoptosis level, and MTT assays indicated that TPAQ and TPAP could promote the cell mortality when cancer cells were in protective autophagy. Furthermore, TPAQ and TPAP could well report the physiological state of cancer cells in autophagy and PDT process in dual‐emissive manner. Particularly, in 3D tumor spheroid chip, the efficient ablation of tumor via the combination of cancer starvation therapy and PDT induced by TPAQ has been successfully confirmed, suggesting the validation of this strategy.

## Conflict of Interest

The authors declare no conflict of interest.

## Supporting information

Supporting Information

## Data Availability

The data that support the findings of this study are available from the corresponding author upon reasonable request.
