# Supplementary material for: Autophagy‐Activated Self‐reporting Photosensitizer Promoting Cell Mortality in Cancer Starvation Therapy
Source: Adv Sci (Weinh). 2023 Apr 21;10(18):2301295. doi: 10.1002/advs.202301295 (PMC10288242; doi:10.1002/advs.202301295)
Supplement: Supplementary file 1 — Supporting Information [file ADVS-10-2301295-s001.pdf]

ADVANCED SCIENCE
Open Access

Supporting Information

for *Adv. Sci.*, DOI 10.1002/advs.202301295

Autophagy-Activated Self-reporting Photosensitizer Promoting Cell Mortality in Cancer Starvation Therapy

Ruoyao Zhang, Chen Zhang, Chao Chen, Minggang Tian, Joe H. C. Chau, Zhao Li, Yuanzhan Yang, Xiaoqiong Li and Ben Zhong Tang**

Supporting Information

Autophagy-activated Self-reporting Photosensitizer Promoting Cell Mortality in Cancer Starvation Therapy

Ruoyao Zhang+,[a], Chen Zhang+,[a], Chao Chen[c], Minggang Tian[d], Joe H. C. Chau[c], Zhao Li[a], Yuanzhan Yang[a], Xiaoqiong Li[a], Ben Zhong Tang*[b]*

[a] School of Medical Technology, Institute of Engineering Medicine, School of Life Science, Beijing Key Laboratory for Separation and Analysis in Biomedicine and Pharmaceuticals, Beijing Institute of Technology, Beijing 100081, China

[b] School of Science and Engineering, Shenzhen Institute of Aggregate Science and Technology, The Chinese University of Hong Kong, Shenzhen, Guangdong 518172, China

[c] Department of Chemical and Biological Engineering and Department of Chemistry, Hong Kong Branch of Chinese National Engineering Research Center for Tissue Restoration and Reconstruction, Division of Life Science and State Key Laboratory of Molecular Neuroscience, The Hong Kong University of Science and Technology, Clear Water Bay, Kowloon, Hong Kong, China

[d] School of Chemistry and Chemical Engineering, University of Jinan, Jinan, Shandong 250022, China

+These authors contributed equally to this work.

Corresponding authors:

Ruoyao Zhang; E-mail: ryzhang@bit.edu.cn;

Ben Zhong Tang; E-mail: tangbenz@cuhk.edu.cn

1. Materials and Methods

1.1 Apparatus and general methods

All the chemicals were purchased and used as received without further purification unless otherwise specified. Nile Red was purchased from J & K. LysoTracker Deep Red (LTDR) and 2', 7'-dichlorodihydrofluorescein diacetate (DCF-DA) were purchased from life technology. The UV-Vis absorption spectra of dilute solutions were recorded on a Milton Roy Spectronic 3000 Array spectrameter using a quartz cuvette with 1 cm path length. Fluorescence spectra were obtained on a Perkin-Elmer LS 55 spectrofluometer. The confocal fluorescent images were obtained with Zeiss LSM 800 confocal laser scanning microscope or Nikon Ti2 confocal laser scanning microscope. The co-localization coefficients and mean fluorescence intensity of the images were determined by the software with Zeiss LSM 800 confocal microscope and Nikon Ti2 confocal microscope.

1.2 Cell culture and staining

Human lung adenocarcinoma cells (A549) and human fetal lung fibroblast-1 (HFL-1) cells were maintained in Roswell Park Memorial Institute 1640 (RPMI 1640) medium supplemented with 10% foetal bovine serum (FBS) and 1% antibiotics (penicillin and streptomycin). Cells were cultured in an incubator with humidified atmosphere of 5% CO_2 at 37°C. Cells were seed into cell culture dish with glass bottom and incubated for overnight.

For live cell staining, A549 cells were treated with 2 μM TPAQ and TPAP for 30 min at 37°C, respectively. For live cell undergoing autophagy, A549 cells were first treated with 2 μM TPAQ and TPAP for 30 min, respectively, and then the complete culture medium was replaced by PBS buffer solution to induce autophagy. For Chloroquine (CQ)-treated cells, A549 cells were first treated with 50 μM CQ, and then treated with 2 μM TPAQ and TPAP for 30 min, respectively, and then the complete culture medium was replaced by PBS buffer solution. For dead cells staining, A549 cells were firstly fixed by 4% PFA for 30 min and then stained with 2 μM TPAQ and TPAP, respectively. For co-staining experiments, A549 cells were stained with 1 μM Nile Red for 15 min

and then incubated with 2 μM TPAQ or TPAP for another 30 min, respectively; A549 cells were treated 2 μM TPAQ or TPAP for 30 min, respectively, then starving for 30 min, and treated with LTDR for another 10 min.

1.3 Theoretical calculation

The frontier orbitals and singlet and triplet energy levels were obtained via density functional theory (DFT) calculation with Gaussian 16 software package. Initially, the geometrical structures of the four molecules were optimized with the basic sets of PM3, B3LYP/6-31G, and B3LYP/TZVP sequentially. The frontier orbitals of the ground state were obtained using the optimized structure by single-point calculation. Afterwards, the geometrical structures of the excited state of the four molecules were optimized via TD-DFT calculation with the basic set of B3LYP/TZVP, and the energy levels of singlet and triplet states were obtained via single-point calculation.

1.4 Cell treatment and MTT assay

For the cell viability detection under normal state, cells were seeded into well plate and incubated overnight. Then cells were treated with different doses of TPAQ and TPAP for 24 h respectively. For the cell viability detection under starved state and light irradiation, cells were firstly treated with TPAQ and TPAP for 1 h respectively. Then, cells in the starvation group were treated with PBS for 2-3 h, and cells in control group were treated fresh complete medium. After starvation, cells in the light group were treated white light irradiation (50 mW/cm^2) for 40 min. Finally, 100 μL basic medium were added into the plate, and then cells both in control group and PBS starvation group (including no light and light group) were incubated at 37℃ for overnight. Cell ability was measured by MTT (5 mg/mL). The optical density readings were detected by a microplate reader at 570 nm. The MTT experiments have repeated at least 3 times.

1.5 TUNEL staining

The apoptosis of A549 cells both in control group and PBS starvation group were measured via TUNEL staining following the manufacturer's instructions (Beyotime). DAPI was used for nuclear staining. Images were captured using confocal microscopy, and the TUNEL-positive cells were counted in at least 5 randomly selected fields of

each group.

1.6 Lysosomal membrane permeabilization detection

The permeabilization of lysosomal membrane of A549 cells after treatment were detected by fluorescent dextran as described previously.[1] In brief, A549 cells were firstly incubated with dextran labeled with Alexa Fluor™ 647 at a concentration of 100 μg/mL (Thermo) for 6-8 h, and then cells were stained with TPAQ and TPAP (20 μM) for 1 h, respectively. After staining, cells were treated with PBS starvation and white light irradiation. After treatment, images of cells were captured using confocal microscopy, and the fluorescence intensity were measured by NIS-Elements software.

1.7 3D cell culture

Here, we have built a 3D cell culture model using microfluidic chip technology according the manufacturer's instructions (MesoBiosystem, China). In brief, A549 cells were mixed with matrix gel in a 1:1 (volume) ratio and then seeded into the bottom chamber of the chip, fresh complete medium was added into the top chamber of the chip for cell culture. After 4-5 days' incubation, A549 cells were stained with TPAQ (20 μM), and then treated with PBS starvation and white light irradiation. Finally, after treatment, images of tumor spheres were captured using confocal microscopy every 24 h. The area of the tumor spheres was measured in at least 5 randomly selected fields of each group at each time point by using NIS-Elements software.

1.8 Statistical analysis

Statistical analysis was performed by using GraphPad Prism software. The data were shown as the mean ± S.E. The difference between two groups was analyzed via two-tailed unpaired Student's t-test, and one-way analysis of variance (ANOVA) followed by Tukey post hoc tests was applied for multiple comparisons. $P < 0.05$ was set as the threshold for significant difference.

2. logP measurement

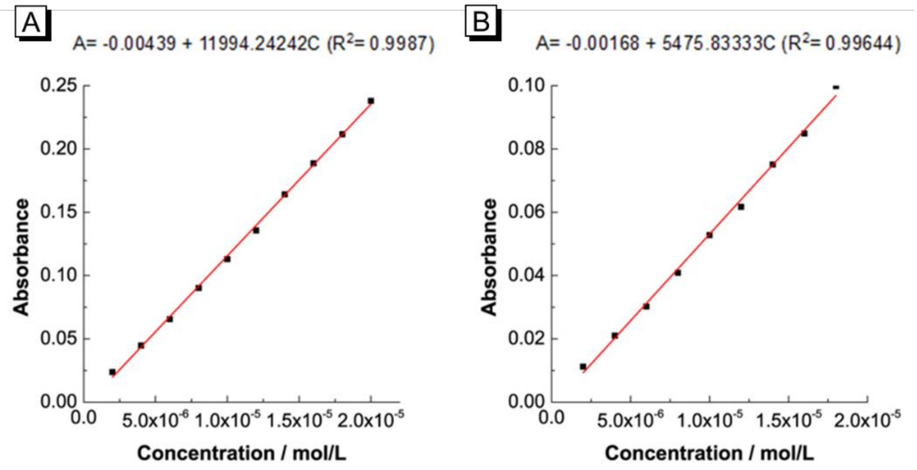

Figure S1. The standard regression equations of compound TPAP in water-saturated *n*-octanol (A) and *n*-octanol-saturated water (B).

Table S1. The *n*-octanol/water partition coefficient of TPAP

Dyes	Concentration (mol/L)	A_o	A_w	C_o (mol/L)	C_w (mol/L)	C_o/C_w	logP
TPAP	3×10^{-5}	0.7017	0.0036	5.89×10^{-5}	9.64×10^{-7}	61.1	1.79

3. Bioimaging

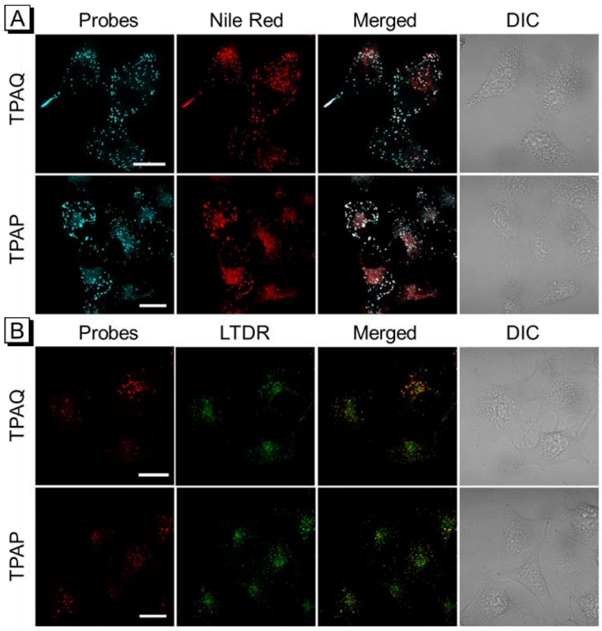

Figure S2. (A) CLSM images of live A549 cells under normal condition stained with 2 μM TPAQ, TPAP, and 1 μM Nile Red, respectively; (B) CLSM images of live A549 cells under starvation condition stained with 2 μM TPAQ, TPAP, and 0.2 μM LTDR, respectively. (A) TPAQ and TPAP: λ_{ex}= 405 nm, λ_{em}= 410-480 nm; Nile Red: λ_{ex}= 561 nm, λ_{em}= 580-650 nm; (B) TPAQ and TPAP: λ_{ex}= 488 nm, λ_{em}= 600-700 nm; LTDR: λ_{ex}= 640 nm, λ_{em}= 650-700 nm. Scale bar = 20 μm.

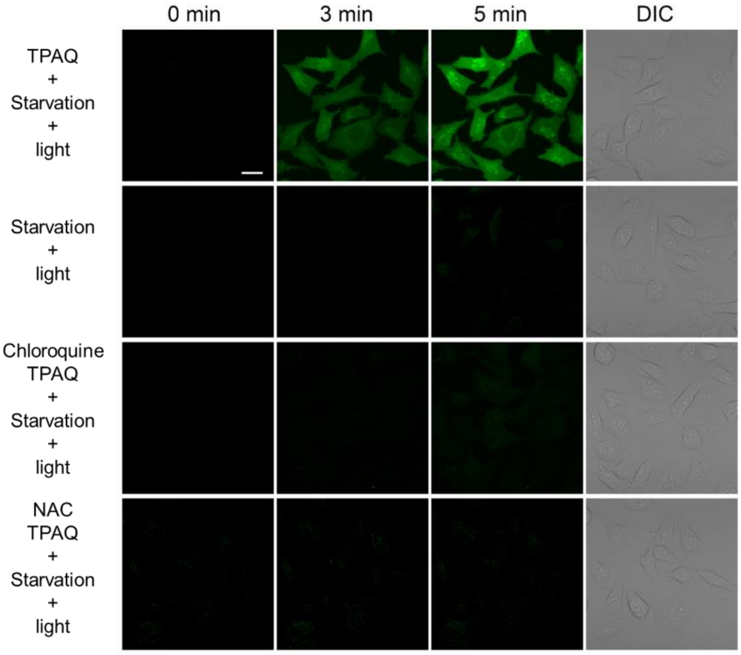

Figure S3. CLSM images of live A549 cells treated with “DCF-DA + TPAQ +

starvation +light", "DCF-DA + starvation + light", "DCF-DA + chloroquine + TPAQ + starvation + light", "DCF-DA + NAC + TPAQ + starvation + light" for different time. λ_{ex}= 488 nm, λ_{em}= 500-600 nm. Scale bar = 20 μm.

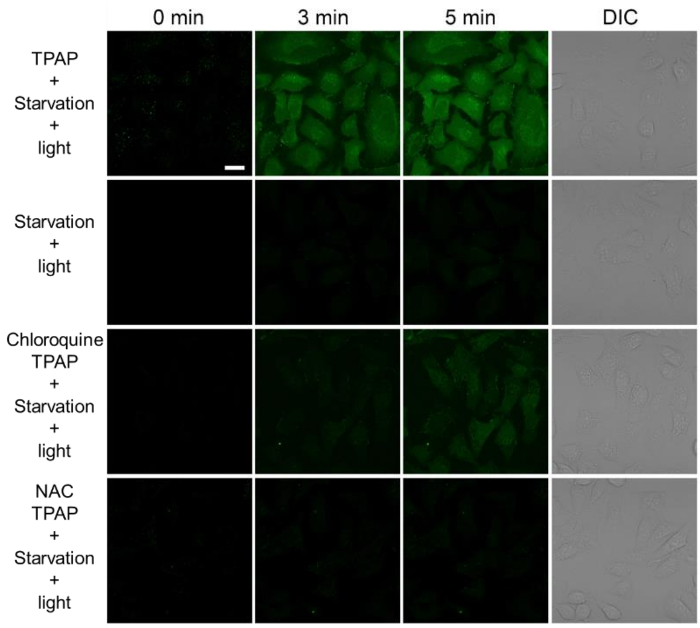

Figure S4. CLSM images of live A549 cells treated with "DCF-DA + TPAP + starvation +light", "DCF-DA + starvation + light", "DCF-DA + chloroquine + TPAP + starvation + light", "DCF-DA + NAC + TPAP + starvation + light" for different time. λ_{ex}= 488 nm, λ_{em}= 500-600 nm. Scale bar = 20 μm.

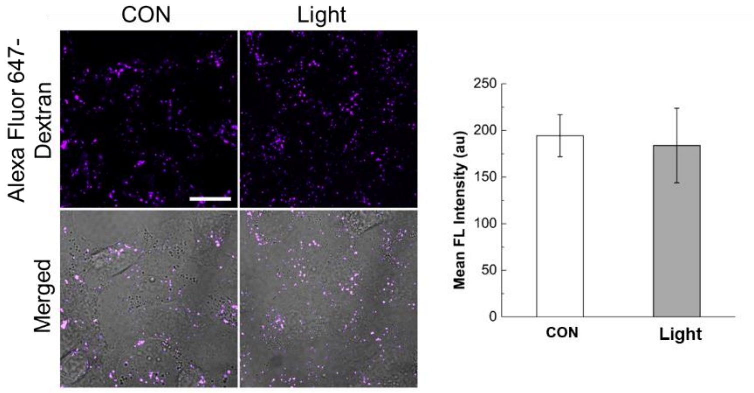

Figure S5. CLSM images of live A549 cells treated with Alexa Fluor 647-Dextran in normal and irradiation conditions, and the relevant mean FL intensity of Alexa Fluor 647-Dextran. λ_{ex}= 640 nm, λ_{em}= 650-720 nm; Scale bar = 20 μm.

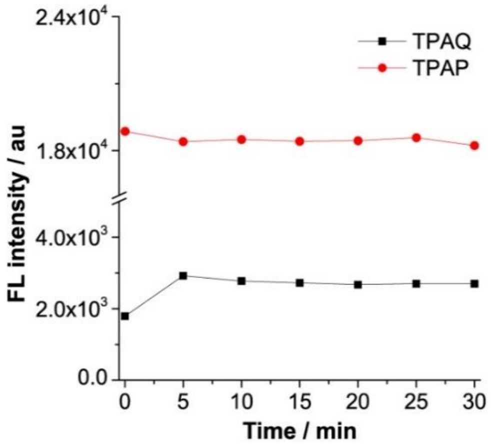

Figure S6. The FL intensity of TPAQ and TPAP in EtOH/PBS (v:v=1:1) solvents (pH = 4.81) with white light irradiation for 30 min. Concentration: 2 μM.

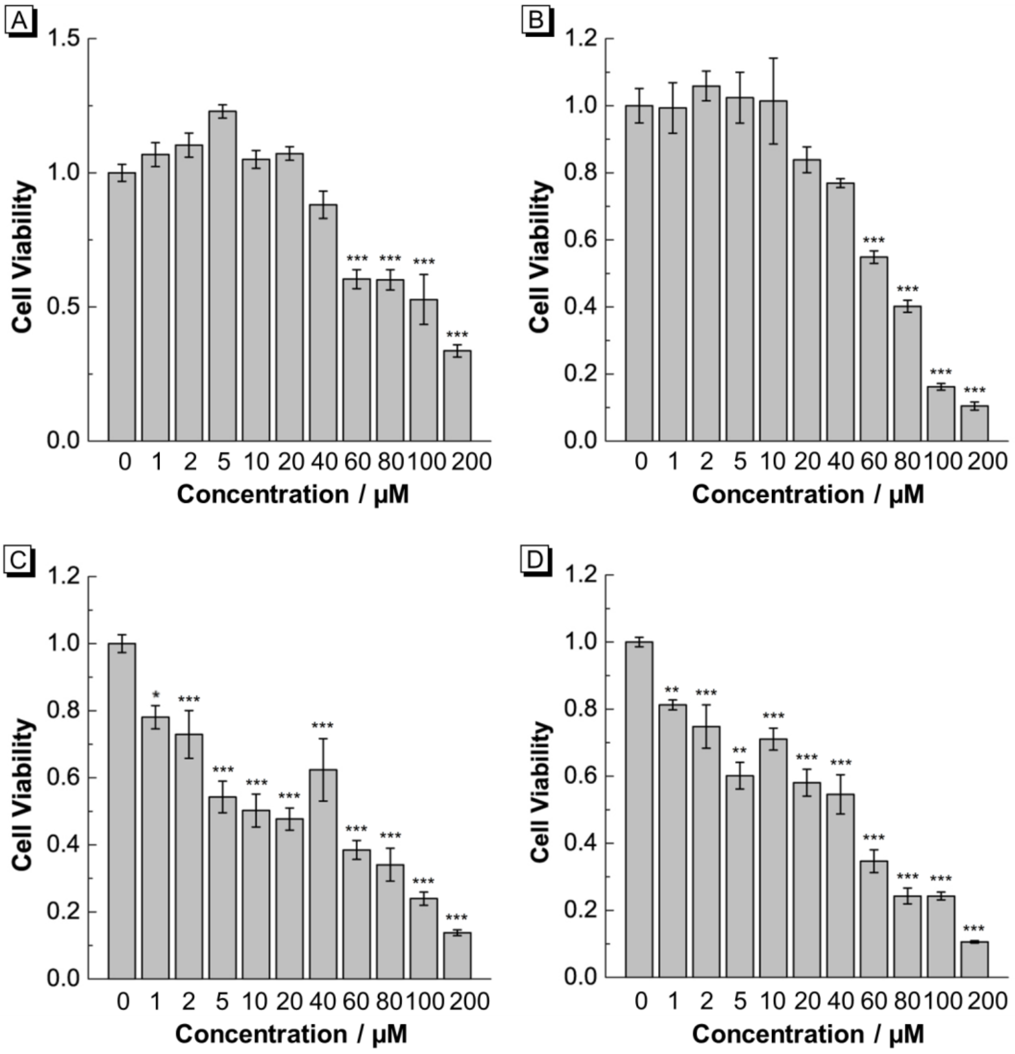

Figure S7. Cell viability of A549 cells stained with TPAQ (A) and TPAP (B) in dark conditions; and cell viability of A549 cells stained with TPAQ (C) and TPAP (D) under

"starvation + light" conditions. Power density: 50 mW/cm^2. *P<0.05, **P<0.01, ***P<0.001 versus control group in one-way analysis of variance (ANOVA) followed by Tukey post hoc tests.

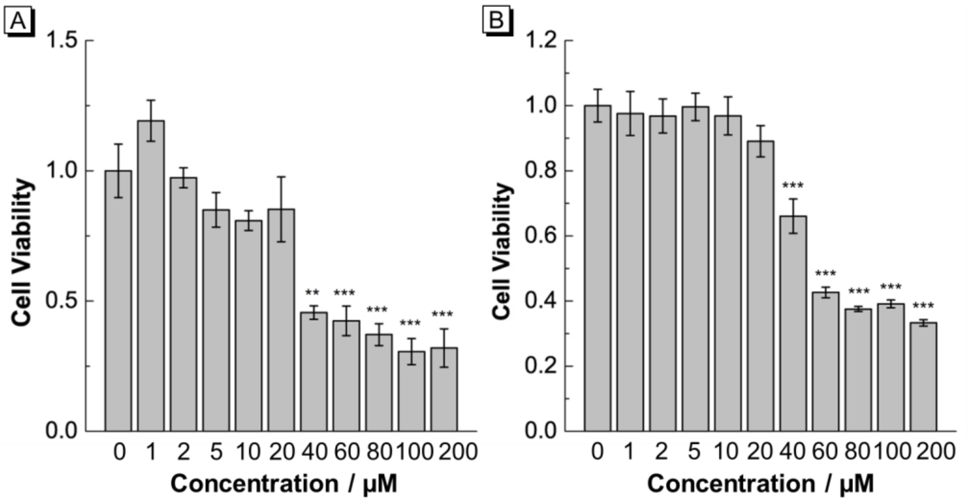

Figure S8. Cell viability of HFL-1 cells stained with TPAQ (A) and TPAP (B) under "normal" conditions. Bar graphs are presented as mean ± S.E. **P<0.01, ***P<0.001 versus control group in one-way analysis of variance (ANOVA) followed by Tukey post hoc tests.

4. Synthetic details, NMR spectra and HRMS spectra

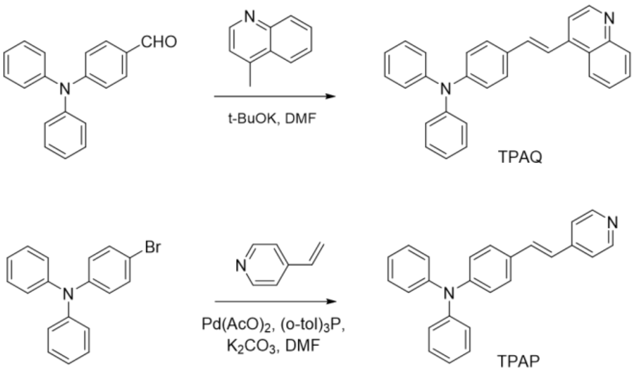

Scheme S1. Synthesis routes of TPAQ and TPAP.

TPAQ and TPAP were synthesized with reference to the published papers.[2,3] The chemical structure of TPAQ has been characterized in our previous work.[2] For TPAP, ^{1}H NMR (400 MHz, DMSO-d_6), δ (ppm): 8.54-8.46 (m, 2H), 7.58-7.45 (m, 5H), 7.38-7.27 (m, 4H), 7.11 (d, J = 7.1 Hz, 2H), 7.08-7.02 (m, 5H), 6.96 (d, J = 8.7 Hz, 2H). ^{13}C NMR (100 MHz, $CDCl_3$), δ (ppm): 150.01, 148.50, 147.28, 145.13, 132.80, 129.78, 129.41, 129.35, 127.99, 124.92, 124.85, 123.86, 123.52, 122.82, 120.66. HRMS m/z: calcd for $C_{25}H_{20}N_2$ 349.1699, found 349.1692 ([M+H]$^+$).

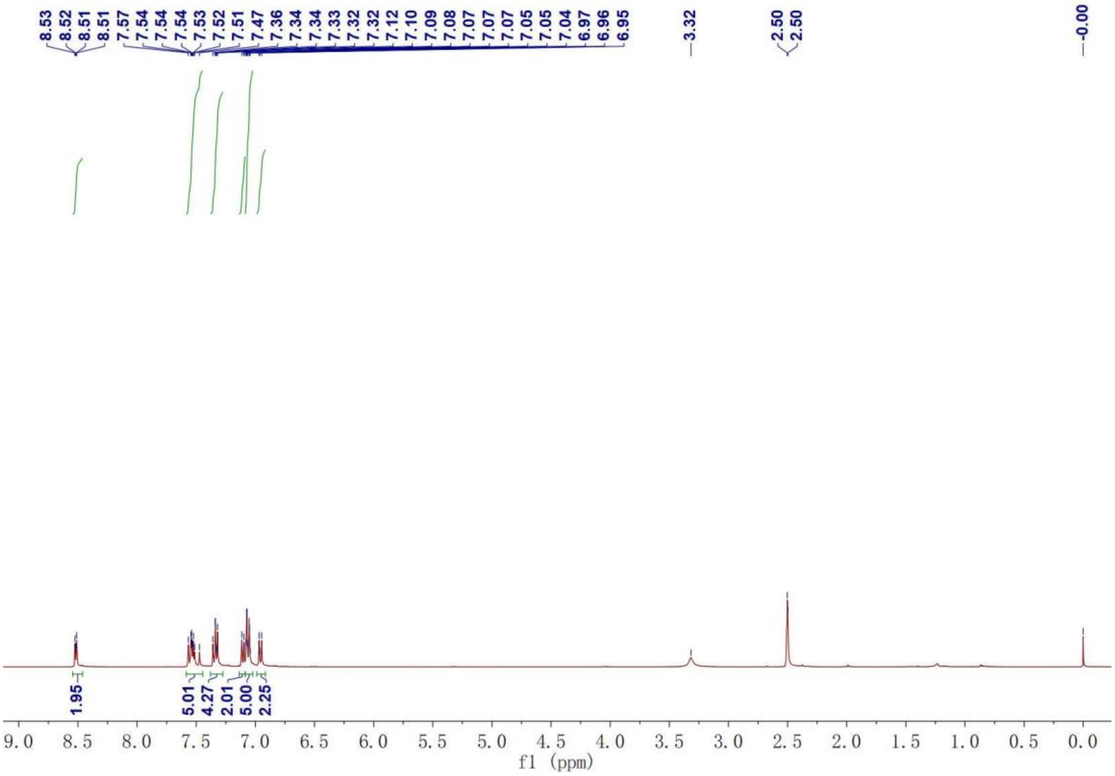

Figure S9. ^{1}H NMR spectrum of TPAP in DMSO-d_6.

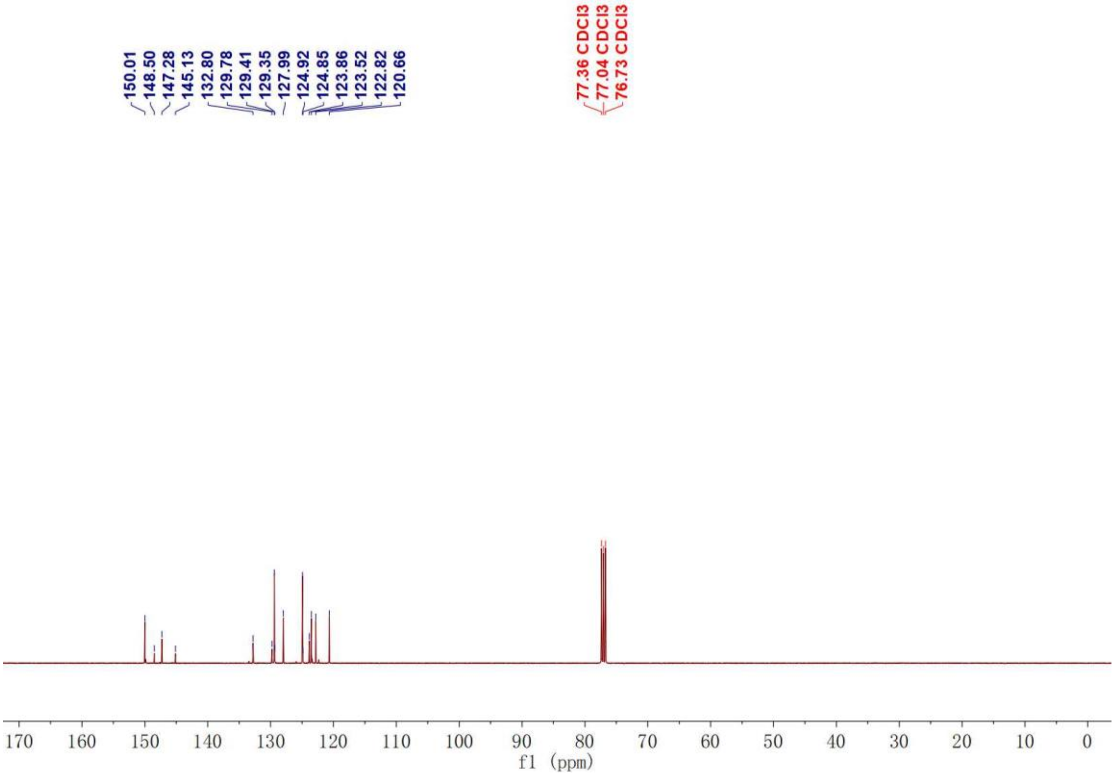

Figure S10. ^{13}C NMR spectrum of TPAP in $CDCl_3$.

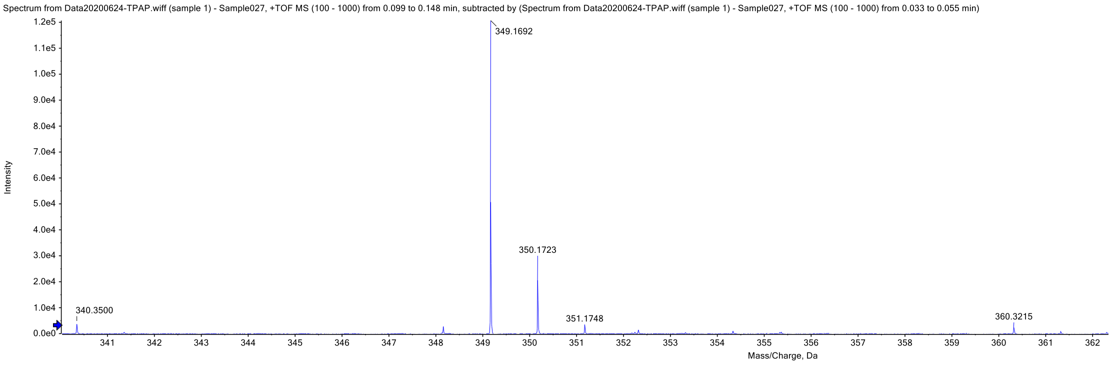

Figure S11. HRMS of TPAP.

5. Reference

[1] A.-M. Ellegaard, M. Jäättelä, J. Nylandsted, *Cold Spring Harb. Protoc.* **2015**, *2015*, 900-903.
[2] R. Zhang, G. Niu, Z. Liu, J. H. C. Chau, H. Su, M. M. S. Lee, Y. Gu, R. T. K. Kwok, J. W. Y. Lam, B. Z. Tang, *Biomaterials* **2020**, *242*, 119924.
[3] M. Tian, Y. Liu, Y. Sun, R. Zhang, R. Feng, G. Zhang, L. Guo, X. Li, X. Yu, J. Z. Sun, X. He, *Biomaterials* **2017**, *120*, 46-56.
